# Effects of Phenytoin on the Retinal Ganglion Cell-Inner Plexiform Layer in Acute Optic Neuritis: Analysis of a Phase II Randomized Trial

**DOI:** 10.1212/WNL.0000000000213951

**Published:** 2025-09-25

**Authors:** Srikirti Kodali, Alessia Bianchi, Rhian Raftopoulos, Marcello Moccia, Anjaneya Prasad Malladi, Marios C. Yiannakas, Magnus Fugger, Rebecca Sara Samson, Claudia Angela Michela Wheeler-Kingshott, Martin Koltzenburg, Ferran Prados, Simon Hickman, Raju Kapoor, Ahmed T. Toosy

**Affiliations:** 1 Department of Neuroinflammation, Queen Square Multiple Sclerosis Centre, UCL Queen Square Institute of Neurology, Faculty of Brain Science, University College London, United Kingdom;; 2 Department of Biomedicine, Neuroscience and Advanced Diagnostic, University of Palermo, Italy;; 3 Kings College Hospital NHS Foundation Trust, London, United Kingdom;; 4 Department of Molecular Medicine and Medical Biotechnology, Federico II University of Naples, Italy;; 5 Multiple Sclerosis Clinical Unit, Federico II University Hospital, Naples, Italy;; 6 Centre for Medical Image Computing, Department of Medical Physics and Biomedical Engineering, University College London, United Kingdom;; 7 e-Health Center, Universitat Oberta de Catalunya, Barcelona, Spain; and; 8 Sheffield Teaching Hospitals NHS Foundation Trust, Sheffield, United Kingdom.

## Abstract

**Background and Objectives:**

Acute demyelinating optic neuritis (AON) can lead to irreversible neuroaxonal loss. We investigated the neuroprotective effects of phenytoin on macular ganglion cell-inner plexiform layer (mGCIPL) thickness.

**Methods:**

We reanalyzed Spectralis optical coherence tomography scans from the phenytoin AON trial (NCT01451593), a randomized, placebo-controlled, double-blind phase 2 trial. Participants attended 2 trial centers in London or Sheffield, United Kingdom. Patients with unilateral AON, age 18–60 years, within 2 weeks of onset, with visual acuities 6/9 or worse, were randomly assigned (1:1) to oral phenytoin (4–6 mg/kg/d) or placebo for 3 months, stratified by onset time, center, previous multiple sclerosis diagnosis, disease-modifying treatment, and corticosteroid use. Macular ganglion cell-inner plexiform layer thicknesses were extracted at baseline and 6 months. Linear regression models evaluated treatment effects on 6-month affected eye mGCIPL, adjusting for 6-month full-field visual evoked potential (VEP) latency and amplitude and baseline variables including mGCIPL thicknesses, steroid use, baseline acuity, and time interval to treatment. We also compared neuroprotective effects between peripapillary retinal nerve fiber layer (pRNFL) and mGCIPL outcomes.

**Results:**

Eighty patients (39 phenytoin: 41 placebo) with a mean age of 33.59 years, 70% female, participated in this study. At 6 months, significant treatment effects were estimated for phenytoin vs placebo (affected eye mGCIPL thicker by 6.79 μm, *p* = 0.006, [SE = 2.35 µm]); estimated means for mGCIPL thickness for phenytoin vs placebo were 73.8 μm (SE = 2.40 μm) and 67.0 μm (SE = 2.2 μm), respectively. Treatment effects appeared to be greater for worse baseline acuities. At 6 months, higher mGCIPL thicknesses were associated with lower VEP latencies (*p* < 0.0001) and higher VEP amplitudes (*p* = 0.013). Neuroprotective effects on mGCIPL outcomes were more robust than for pRNFL outcomes.

**Discussion:**

This study supports superiority of the mGCIPL over the pRNFL as a neuroprotective marker after AON and demonstrates strong associations with myelination, providing additional mechanistic insights.

**Trial Registration Information:**

This trial is registered with ClinicalTrials.gov, number NCT01451593; submitted for registration on October 11, 2011; first patient enrollment was on February 2, 2012.

**Classification of Evidence:**

This study provides Class II evidence that compared with placebo, phenytoin is associated with greater preservation of the mGCIPL thickness in patients with acute demyelinating optic neuropathy.

## Introduction

Multiple sclerosis (MS) is an autoimmune demyelinating disease of the CNS which can result in accumulating disability.^1^ The focus of treatments for MS is to halt disability progression through immune modulation. Disability accrual results from both accumulation of residual deficits after relapses and chronic progression independent of relapses.^2^ Recently, therapeutic remyelination^3,4^ and neuroprotection^5^ trials have been undertaken in the acute/early phase of disease to prevent further disability in addition to traditional immunomodulatory therapies.

Up to 70% of people with MS experience acute optic neuritis (AON) during their disease course. AON is caused by an acute inflammatory, demyelinating lesion in the optic nerve, histologically identical to MS plaques in the CNS.^6,7^ AON is an excellent model to study MS relapses, allowing the evaluation of neuroprotective and remyelinative therapies.^8^ Structural changes to the optic nerve after AON described through optical coherence tomography (OCT), correlate, in vivo, with quantifiable functional measures for example, visual evoked potentials (VEPs), measurements of high/low contrast letter visual acuity (VA) and color vision, providing valuable pathobiologic insights.

OCT can track neuronal and axonal integrity in MS^6^ through peripapillary retinal nerve fiber layer (pRNFL) and ganglion cell inner plexiform layer (GCIPL) thickness measurements. GCIPL is a proxy for measuring the integrity of the retinal ganglion cell layer. This contains cell bodies that project axons into the pRNFL and which converge on the optic nerve head. pRNFL measures can be confounded by optic nerve head swelling in early AON. GCIPL is less affected by inflammation.^9^

The phenytoin AON study was a landmark, randomized, placebo-controlled phase II trial^10^ that evaluated the neuroprotective potential of selective blockade of voltage-gated sodium channels. Oral phenytoin (4–6 mg/kg/d) or placebo was administered for 3 months, starting within 2 weeks of symptom onset. Participants were then followed up to 6 months. The original study found a significant difference in affected eye mean 6-month pRNFL thickness between groups, with the phenytoin group showing 30% greater preservation of pRNFL thickness.

Macular ganglion cell-inner plexiform layer (mGCIPL) thickness has been suggested as a superior outcome to pRNFL thickness in measuring early atrophy after AON.^9^ The primary research questions addressed in this study were (1) does phenytoin treatment have a neuroprotective effect on mGCIPL; (2) is mGCIPL superior to pRNFL as a marker of neuroprotection and can this validated using association with electrophysiologic parameters; and (3) does severity of baseline visual impairment affect treatment effects? A greater understanding of mGCIPL thickness through addressing these questions would inform its utility for future neuroprotection trials.

## Methods

### Study Design and Participants

**Figure 1:**
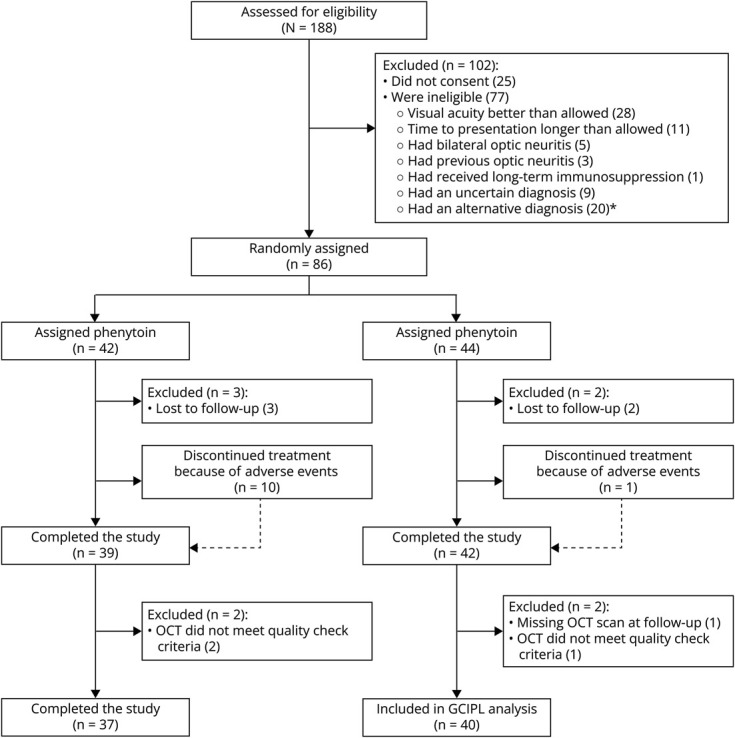
Phenytoin GCIPL Study. Modified from Original Phenytoin Trial Patient Flow Diagram *Alternative diagnoses were functional visual loss (n = 4), sarcoidosis (n = 3), migraine with aura (n = 2), posterior scleritis (n = 2), Leber hereditary optic neuropathy (n = 2), compressive optic nerve lesions (n = 2), uveitis (n = 1), toxic optic neuropathy (n = 1), neuroretinitis (n = 1), central serous retinopathy (n = 1), and optic nerve drusen (n = 1). GCIPL = ganglion cell-inner plexiform layer.

The phenytoin AON trial (NCT01451593) study protocol has been previously outlined.^10^ Participants attended 2 trial centers in London or Sheffield, United Kingdom. Eligible candidates were age 18–60 years, with a clinical diagnosis of unilateral AON (confirmed by a neuro-ophthalmologist) without a previous history of clinical AON in either eye, a VA 6/9 or worse in the affected eye and were within 14 days of disease onset before randomization. Inclusion and exclusion criteria, as well as phenytoin dosing and duration, have been presented in the main study report.^10^ In total, 80 patients were randomized, 1:1, to oral phenytoin or placebo (Figure 1). Corticosteroid use was at the treating physician's discretion and was stratified between each group. In this study, we conducted new analyses on the participants' OCT scans and electrophysiologic and visual outcomes.

### Study Procedures

#### OCT

At baseline and 6 months, pRNFL and macular scans were acquired with a high-resolution spectral-domain OCT platform (Spectralis, Heidelberg Engineering, Germany; Software V 5.4B). The pRNFL scans were acquired from 3.45-mm diameter ring around the optic nerve head, and fast macular volume scans were acquired from a 20 × 20° field with 25 horizontal B scans and an automatic real time of 9. OSCAR-IB quality control criteria were followed and scans with a signal strength of <25 was excluded.^11^ mGCIPL average thickness was derived between 1 and 3 mm eccentricity on the 1-2-3 mm concentric ring grid centered on the fovea.^11^ The macular OCT scans underwent automated segmentation on the Spectralis OCT platform. The segmented layers were inspected and manually adjusted by a single rater (S.K.) using the following quality control criteria: marked decentration, significant segmentation failure because of poor signal-to-noise ratio, macula quality <25, presence of other obvious retinal pathology, and poor illumination.

#### Visual Clinical Outcomes

Best-corrected high-contrast logMAR VA was measured using retro-illuminated Early Treatment Diabetic Retinopathy Study charts at 4 m. A score of 1.7 was assigned by the masked researcher when no letters could be correctly identified.

#### VEPs

Full-field VEPs to reverse achromatic checks were recorded at baseline and 6 months. These were acquired according to International Federation of Neurophysiology guidelines on a Synergy system (Viasys Healthcare, Conshokocken, PA) in standard background office lighting. Responses were recorded from the occipital midline (Oz), using midline frontal (Fz) as reference and midline central (Cz) as ground. Patterned stimuli were defined by the visual angle subtended by the side of a single check (large check = 1°, small check [SC] = 0.25°) from the eye. Small check latency and amplitude of the P100 component were measured to 1 decimal place in the replicates.

### Statistical Analysis

Our primary objective was to investigate effects of phenytoin treatment on OCT structural measures in AON, 6 months after onset of visual loss. We used linear regression models to evaluate structural OCT parameters, demographic characteristics, visual clinical outcomes, and VEP parameters between treatment and placebo groups. Analysis was performed on a modified intention-to-treat population of all randomized participants who had both baseline and 6-month scans.

#### Primary Analysis: Treatment Effect on mGCIPL

We initially constructed a linear regression initial model with affected eye 6-month mGCIPL thickness as the outcome variable and the following predictors: treatment allocation, unaffected and affected eye baseline mGCIPL thickness, age, sex, and recruitment center.

To improve the precision of the basic model, we added additional predictor variables, 1 at a time in a nested fashion. These additional variables included days between symptom onset and OCT (which coincided with treatment onset), time between commencement of corticosteroids and OCT (no vs 1–5 days vs 6–30 days), affected eye 6-month small-check N75-p100 amplitude and p100 latency, and affected eye baseline VA. We excluded participants with unrecordable affected eye VEP responses at 6 months. Models were compared with standard model performance criteria using Akaike information criterion corrected (AICC), Bayesian information criterion (BIC), coefficient of determination (*R*^2^), and root mean square error (RMSE) to establish the best fit model that predicted treatment effect on mGCIPL thickness at 6 months. The best fit final model included the following predictors: baseline unaffected eye mGCIPL thickness; demographic variables; recruitment center; electrophysiologic variables (VEP amplitude, latency); time between symptom onset and baseline assessment; time between corticosteroid administration (if any) and baseline assessment; and visual loss severity at baseline.

#### Post Hoc Analysis

We investigated whether the treatment effect on 6-month mGCIPL thickness varied as a function of baseline affected eye VA by adding an interaction term to the final model (treatment × baseline VA). This allowed us to determine treatment effects at different levels of baseline acuity.

#### Secondary Analyses: Comparing Treatment Effects on mGCIPL vs pRNFL

We compared phenytoin treatment effect on 6-month affected eye mGCIPL and pRNFL thicknesses as alternative outcomes, using standard model performance metrics: adjusted *R*^2^, BIC, AICC, and RMSE. The predictors for each model were identical except unaffected and affected baseline pRNFL were swapped for unaffected and affected baseline mGCIPL for the pRNFL outcome model.

#### Sensitivity Analyses

We performed sensitivity analyses for the outcome 6-month affected mGCIPL thickness, by investigating the following predictors.

##### Affected Eye Baseline VA vs Use of Corticosteroids

Corticosteroid use in AON is usually related to severity of baseline visual loss. Although participants receiving corticosteroids were matched between placebo and treatment groups, we performed an analysis where corticosteroid use replaced baseline affected eye VA in the final model.

##### VEP Variables

We explored associations between mGCIPL and treatment effect with respect to the contribution of 6-month electrophysiologic variables (P100 latency, N75-P100 amplitude) as follows.

1. We removed electrophysiologic variables from the final model to determine whether treatment effects were preserved.
2. We replaced small-check size VEP predictors in the final model (P100 latency, N75-P100 amplitude) with large-check VEP predictors and compared model performances.
3. We compared the final model with a similar one that included observations with absent 6-month VEP responses (assigned as 200msec latencies and 0 μV).

Analyses were performed using Rstudio, version 2023.06.1 + 524 software (Softonic International, Barcelona, Spain). All tests were 2-tailed, and statistical significance was considered when *p* values were <0.05.

### Standard Protocol Approvals, Registrations, Patient Consents

All participants gave written informed consent before entry. The study was approved by the London-Southeast United Kingdom Research and Ethics Committee on November 15, 2011. The trial was registered with ClinicalTrials.gov, number NCT 01451593, and the full protocol is available online and in the original paper for reference.^10^

### Data Availability

Anonymized participant data can be shared on reasonable request to the corresponding author by a qualified investigator for the purposes of replicating procedures and results.

## Results

Eighty participants were recruited from February 2012 to May 2014. Thirty-nine participants were randomized to phenytoin and 41 to placebo (Figure 1). In the primary clinical trial publication, the authors analyzed a total of 81 participants (39 in phenytoin group, 42 in the placebo group). Of these 81 participants, 1 from the placebo group had a missing OCT macular scan at 6 months and was therefore excluded in this analysis. This was also acknowledged in the original trial paper.^10^ Three further patients were excluded because of poor quality macular OCT scans; however, they were distributed across both groups. Of the remaining 77, 10 more were excluded from the final model because of unrecordable VEP responses at 6 months. Both groups exhibited similar baseline characteristics (Table 1).

**Table 1:** Baseline and 6-Month Raw (Unadjusted) Summary Characteristics of the Participants

	Phenytoin (n = 37) mean (SD)	Placebo (n = 40) mean (SD)	T-value or χ² (df) *p* value
Age (y)	32.85 (7.69)	34.28 (9.33)	−0.74 (74.05) *p* = 0.46
Percentage who were female, (%)	68	72.5	0.29 (1) *p* = 0.59
Time between symptom onset and assessment (d)	7.89 (2.98)	8.38 (3.10)	−0.70 (74.88) *p* = 0.48
Time between symptom onset and corticosteroids–if given (d)	7.90 (3.69)	7.93 (3.10)	−0.04 (70.58) *p* = 0.97

Baseline				
	Phenytoin		Placebo	
	Affected	Unaffected	Affected	Unaffected
mGCIPL thickness (µm)	92.47 (8.51)	92.26 (7.97)	88.83 (7.92)	90.94 (7.32)
pRNFL thickness (µm)	123.78 (44.18)	101.78 (21.71)	107.88 (35.32)	99.20 (12.00)
N75-P100 amplitude SC (µV)	2.35 (3.76)	9.74 (5.32)	3.08 (3.84)	10.70 (5.88)
P100 latency SC (ms)	172.28 (33.70)	106.52 (17.06)	165.84 (36.36)	104.61 (6.00)
logMAR VA	1.12 (0.57)	−0.05 (0.09)	1.00 (0.62)	−0.08 (0.09)

6 mo				
	Phenytoin		Placebo	
	Affected	Unaffected	Affected	Unaffected
mGCIPL thickness (µm)	71.29 (13.86)	91.19 (8.92)	65.19 (13.01)	89.44 (11.92)
pRNFL thickness (µm)	79.86 (17.77)	98.03 (11.65)	75.03 (17.57)	96.65 (14.22)
N75-P100 amplitude SC (µV)	6.29 (4.68)	8.42 (5.24)	7.03 (4.87)	9.55 (6.37)
P100 latency SC (ms)	139.59 (30.28)	115.69 (31.86)	130.66 (25.07)	110.78 (21.64)
logMAR VA	0.17 (0.42)	−0.02 (0.17)	0.10 (0.22)	−0.06 (0.12)

Abbreviations: mGCIPL = macular ganglion cell-inner plexiform layer; pRNFL = peripapillary retinal nerve fiber layer; SC = small check; VA = visual acuity.

Data are mean (SD) or number (%).

VEP statistics (N75-P100 amplitude SC, P100 latency SC) include absent VEPs where 0 µV and 200 ms were assigned.

### Primary Analysis

**Figure 2:**
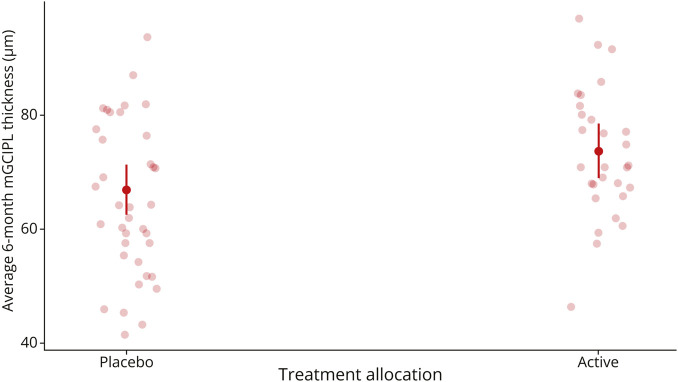
Estimated 6-Month Affected Eye mGCIPL Thickness

The final model estimated a significantly higher mGCIPL thickness by 6.79 µm (*p* = 0.006, standard error [SE] = 2.35 µm) in the 6-month affected eyes of participants receiving phenytoin treatment compared with placebo (n = 67). Adjusted means for treatment and placebo groups were 73.8 µm (SE = 2.40 µm) and 67.0 µm (SE = 2.21 µm), respectively, averaged across other predictors (Figure 2).

In addition to treatment effect, the following predictors were also significant for 6-month mGCIPL thickness: baseline affected logMAR VA (β = −5.54 µm, *p* = 0.010, SE = 2.08 µm), P100 SC latency (β = −0.32 µm, *p* < 0.001, SE = 0.07 µm), and N75-P100 SC amplitude (β = 0.78 µm, *p* = 0.013, SE = 0.30 µm). The following predictor variables were not significant: age (β = −0.03 µm, *p* = 0.85, SE = 0.14 µm), sex (β = 3.05 µm, *p* = 0.30, SE = 2.92 µm), center (β = 1.61 µm, *p* = 0.64, SE = 3.39 µm), time from visual loss to assessment (β = 0.13 µm, *p* = 0.73, SE = 0.36 µm), and time from corticosteroid prescription to assessment (β = 0.49, *p* = 0.86, SE = 2.84 µm).

**Figure 3:**
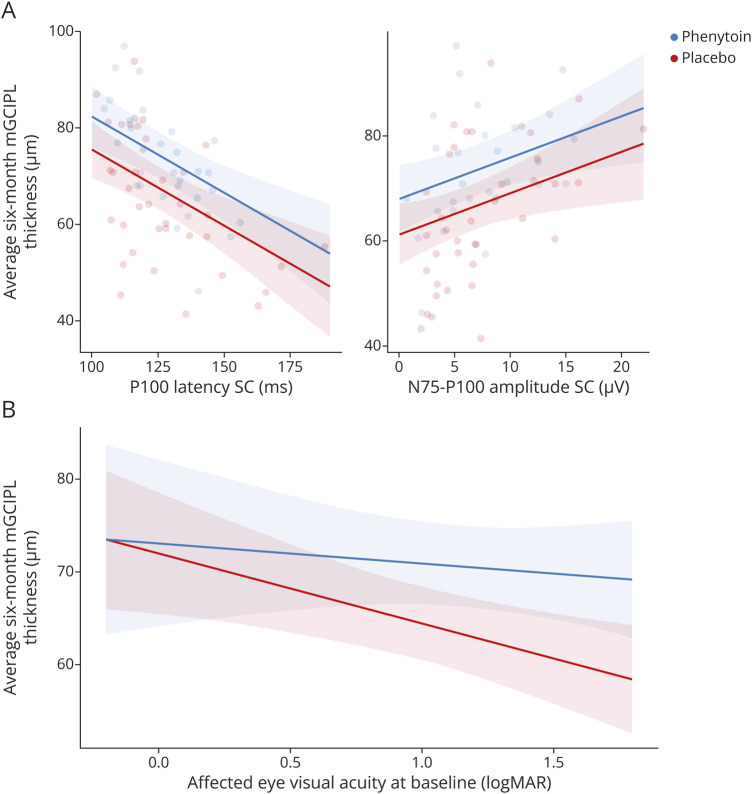
Predictive Plot of 6-Month Affected Eye mGCIPL Thickness (A) Predictive plots of macular ganglion cell-inner plexiform layer (mGCIPL) thickness values compared with p100 small check latencies and N75 P100 small check amplitudes for treatment and placebo groups. (B) Treatment effect on predicted mGCIPL estimates for various baseline visual acuities. mGCIPL = macular ganglion cell-inner plexiform layer; SC = small check.

The model predicted that a 1-ms increase in 6-month VEP latency was associated with a 0.32 µm (SE = 0.07 µm, *p* < 0.001) reduction in 6-month mGCIPL thickness and that a 1-µV increase in VEP p100 amplitude was associated with a 0.78-µm (SE = 0.30 µm, *p* = 0.013) increase in mGCIPL thickness (Figure 3).

#### Post Hoc Analysis

We investigated whether treatment effect on 6-month mGCIPL was dependent on baseline vision by their interaction. We report predicted slopes for placebo and treatment groups (Figure 3B). Although the interaction between slopes was not significant (β = 5.37 µm/logMAR unit, *p* = 0.230, SE = 4.42 μm/logMAR unit), the placebo slope was significantly different to zero (β = −7.52 μm/logMAR unit, *p* = 0.0062, SE = 2.64 μm/logMAR unit), whereas the phenytoin slope was not (β = −2.15 μm/logMAR unit, *p* = 0.5280, SE = 3.47 μm/logMAR unit) implying that patients treated with phenytoin were more likely to preserve their mGCIPL. This informed further estimation of marginal treatment effects at different values of baseline affected eye vision, which revealed a logMAR VA threshold ≥0.775 (Table 2), above which phenytoin effects were beneficial compared with placebo (approximately 20/120 Snellen equivalent or worse).

**Table 2:** Treatment Effect for mGCIPL Thickness Outcomes of Phenytoin on Macular Ganglion Cell-Inner Plexiform Layer Thickness for Different Baseline Affected Eye Visual Acuities

logMAR VA	Estimated treatment effects (µm)	SE	*p* Value
0.00	1.14	5.20	0.8275
0.5	3.82	3.38	0.2632
0.6	4.36	3.08	0.1624
0.7	4.90	2.81	0.0873
0.75	5.17	2.70	0.0607
0.77	5.27	2.65	0.0520
0.775	5.30	2.64	0.0500^a^
0.8	5.43	2.59	0.0409^a^
1.0	6.51	2.35	0.0078^b^
1.5	9.19	3.07	0.0041^b^
1.7	10.27	3.20	0.0076^b^

Abbreviations: mGCIPL = macular ganglion cell-inner plexiform layer; SE = standard error; VA = visual acuity.

a 0.01 < *p* ≤ 0.05.

b *p* ≤ 0.01.

### Secondary Analyses: Comparing mGCIPL Versus pRNFL

An adjusted increase of 6.93 µm (*p* = 0.034, SE = 3.18 µm) for 6-month affected eye pRNFL thickness was observed with phenytoin treatment compared with placebo. In addition, the following variables significantly predicted 6-month pRNFL thickness: baseline affected eye logMAR VA (β = −9.82 µm, *p* = 0.002, SE = 3.02 µm), unaffected eye baseline pRNFL thickness (β = 0.4617, *p* < 0.001, SE = 0.09 µm), and P100 SC latency (β = −0.26 µm, *p* = 0.016, SE = 0.10 µm). The following predictor variables did not significantly predict pRNFL thickness: age (β = −0.14 µm, *p* = 0.49, SE = 0.20 µm), sex (β = −5.55 µm, *p* = 0.18, SE = 4.10 µm), center (β = −3.36 µm, *p* = 0.48, SE = 4.76 µm), N75-P100 SC amplitude (β = 0.51 µm, *p* = 0.23, SE = 0.42 µm), time from visual loss to assessment (β = −0.17 µm, *p* = 0.74, SE = 0.52 µm), and time from corticosteroid prescription to assessment (β = 3.56 µm, *p* = 0.37, SE = 3.96 µm).

Based on adjusted *R*^2^, BIC, AICC and RMSE comparisons, the mGCIPL model was more robust in reflecting the effects of treatment (e.g., RMSE for mGCIPL thickness was 7.98 vs 11.26 for the pRNFL thickness model). In addition to these metrics, mGCIPL thickness was strongly associated with VEP latency and amplitude, whereas pRNFL was only associated with latency not amplitude, substantiating mGCIPL as a physiologically more meaningful structural outcome measure than pRNFL thickness.

### Sensitivity Analyses for the mGCIPL Model

Sensitivity analyses explored the relationship between mGCIPL thickness and severity of AON. Corticosteroid use was associated with worse baseline VA (β = 0.7877 logMAR units, *p* < 0.001, SE = 0.15 logMAR units). Corticosteroid use was also randomized between placebo and treatment groups (*p* = 0.77). We compared our final primary analysis model with a similar model, replacing baseline affected eye VA with corticosteroid use. Model performance was better with the original model, that is, with the use of baseline VA.
(i) When we removed the VEP variables completely, treatment effects remained significant (from *p* = 0.0056 [β = 6.79 µm, SE = 2.35 µm] to p = 0.0306 [β = 6.35 µm, SE = 2.86 µm]). (ii) Treatment effects were more significant with small vs large check latency and amplitude values (*p* = 0.0056 [β = 6.79 µm, SE = 2.35 µm] vs *p* = 0.0347 [β = 5.43 µm, SE = 2.51 µm, respectively). SC variables as predictors were associated with better model performance (lower RMSE with SC compared with large check [7.977 vs 8.302, respectively]). (iii) We also compared 2 models that included/excluded absent VEP responses. The final model reported above, excluded absent VEP responses (treatment effect *p* = 0.0056) and exhibited a better model fit than including absent VEP responses (RMSE for excluded absent VEPs = 7.977 vs RMSE for included absent VEPs = 9.499). Treatment effects remained significant when absent VEP variables were included (*p* = 0.0042, β = 7.30 µm, SE = 2.56 µm).

### Classification of Evidence

This study provides Class II evidence that compared with placebo, phenytoin is associated with greater preservation of the macular ganglion cell-inner plexiform layer thickness in patients with acute demyelinating optic neuropathy.

## Discussion

In this analysis of a phase 2 clinical trial, phenytoin treatment was associated with significant preservation of mGCIPL thickness after AON, compared with placebo. These results corroborate the original trial findings,^10^ where the primary outcome was pRNFL thickness. Our results are consistent with the suggestion that phenytoin protects retinal ganglion cells and their axons in the optic nerve through partial inhibition of voltage-gated sodium channels and related metabolic changes after acute focal inflammatory demyelination. Our results support the mGCIPL as a more reliable marker of neuroaxonal integrity in AON where the effect of inflammation or disc swelling can confound pRNFL measurement.

Sodium channels are expressed at high density in myelinated axons. The inflammatory demyelinating lesion of AON can induce retrograde and anterograde degeneration. Retrograde degeneration can manifest as loss of retinal ganglion cells. As mGCIPL thickness was preserved in our analysis, we infer that phenytoin, by inhibiting the increase of intracellular sodium, can ameliorate retrograde degeneration.

We also report a significant association between mGCIPL thickness and optic nerve function, as measured by VEP amplitude and latency. This suggests that preserved mGCIPL thickness with phenytoin treatment is associated with retention of functional myelinated optic nerve fibers. Although the cohort size was not substantial, this study was powered adequately based on previously calculations from AON OCT longitudinal studies.^8,10^

We investigated the effect of affected eye baseline VA on phenytoin treatment effect and inferred greater neuroprotective effects in eyes with worse baseline visual acuities. This suggests that selective sodium channel blockade is effective when the optic nerve experiences greater acute functional impairment, perhaps reflecting greater neuronal energy failure, leading to reduced activity of the membrane sodium-potassium ATPase. Significant treatment effects were seen with visual acuities ≥0.775 logMAR. This could potentially be used to inform treatment decisions for phenytoin if it becomes available for neuroprotection in AON in future.

Phenytoin treatment also significantly preserved pRNFL thickness in our analysis,^10^ but we inferred that mGCIPL thickness is a more robust structural marker of neurodegeneration to assess the neuroprotective effect of phenytoin in AON.^12^ This can be explained statistically by larger standardized metrics of effect sizes for mGCIPL compared with pRNFL thickness (for mGCIPL: standardized coefficient = 0.52 [95% CI 0.16–0.89], partial eta-squared = 0.20 vs for pRNFL: standardized coefficient = 0.40 [95% CI 0.03–0.77], partial eta-squared = 0.11), accompanied by smaller variability (SE = 2.35 vs 3.18). Moreover, mGCIPL thickness had stronger and significant associations with optic nerve function, as measured by VEP amplitude and latency making it a more functionally relevant structural measure. The treatment effect remained robust to different VEP parameters. Small check VEP variables produced better model fits than large check. It would be interesting to look at the treatment effects on ganglion cell layer and IPL thickness individually although it is open question whether ganglion cell layer and IPL segmentation is reliable after automated segmentation and might be difficult to distinguish with naked eye. Effects of neuroprotection on the retinal inner nuclear layer and microcystic macular edema, which are emerging markers of inflammation, might be interesting to study in future analysis.

Regarding potential sources of bias, the clinical, structural, and electrophysiologic characteristics of the placebo and phenytoin groups at baseline were similar and typical of patients with AON. The loss of pRNFL thickness in the placebo group at 6 months was consistent with previous natural history studies of AON.^13,14^ Care was taken to exclude patients with atypical AON (none of the participants had serum aquaporin-4 antibodies), and no participants developed features of disorders such as neuromyelitis optica or chronic relapsing inflammatory optic neuropathy. Testing for antibodies to myelin oligodendrocyte glycoprotein (another immunologic subtype of AON) was not available at the time of the trial and therefore was not performed. This and any further characterization of immunologic subtypes of AON could be tested in future trials to understand the differences in their response to neuroprotective therapies.

One participant was on disease-modifying treatment before month 6. We did not use being on treatment as a binary covariate and followed a similar statistical methodology to the original trial publication.^10^ Refractive error, particularly myopia, can confound mGCIPL measurements. One study^15^ showed this association in young Chinese adults and found myopic eyes to have a thinner mGCIPLs compared with normal eyes. There is no clear consensus on whether severe refractive error should be a rejection criterion especially for the OSCAR-IB criteria.^16^ Detailed refraction data were not systematically recorded for this study or for the original phenytoin study. Future studies should investigate the impact of refractive error on mGCIPL and pRNFL.

At baseline, on average, pRNFL swelling was noted in the affected eye. However, mGCIPL is not affected by swelling. Interestingly, mean baseline affected eye mGCIPL thickness for the treatment arm was slightly higher than the placebo arm (*p* = 0.056 [SE = 1.91 µm] and Table 2). We correspondingly adjusted for both baseline affected and unaffected eye mGCIPL and pRNFL thicknesses in the regression analyses to account for any confounding effects.

Corticosteroid treatment at presentation is unlikely to have affected the findings of this study. Participants receiving corticosteroids were randomized between the phenytoin and placebo groups. Corticosteroids are considered for severe visual loss at presentation. The use and timing of corticosteroid administration after symptom onset was adjusted for in the statistical analysis. In addition, we adjusted for the severity of AON by including the presenting affected eye VA as a predictor variable. Previous studies have shown that high-dose corticosteroid treatment does not prevent pRNFL thinning^13^ or optic nerve atrophy^17^ or improve visual outcomes after MS-related AON.^13,15,17,18^

In conclusion, the results of this study extend the original phenytoin trial results for the neuroprotective effect of phenytoin in AON. Our analysis recommends the use of mGCIPL thickness measurement as a more robust and physiologically relevant marker of neurodegeneration in AON compared with pRNFL thickness. Phenytoin has greater benefit on mGCIPL outcomes when early visual acuities are worse, reinforcing the clinical relevance of mGCIPL. Therefore, mGCIPL thickness measurement should be preferentially considered as an outcome measure in trials of neuroprotection in AON.

## Glossary

AICC: Akaike information criterion corrected
AON: acute optic neuritis
BIC: Bayesian information criterion
GCIPL: ganglion cell inner plexiform layer
mGCIPL: macular ganglion cell-inner plexiform layer
MS: multiple sclerosis
NIHR: National Institute for Health Research
OCT: optical coherence tomography
pRNFL: peripapillary retinal nerve fiber layer
RMSE: root mean square error
SC: small check
SE: standard error
VA: visual acuity
VEP: visual evoked potential
